# Isolation of *Candidatus* Rickettsia vini from Belgian *Ixodes arboricola* ticks and propagation in tick cell lines

**DOI:** 10.1016/j.ttbdis.2020.101511

**Published:** 2020-11

**Authors:** Alaa M. Al-Khafaji, Lesley Bell-Sakyi, Gerardo Fracasso, Lisa Luu, Dieter Heylen, Erik Matthysen, José A. Oteo, Ana M. Palomar

**Affiliations:** a Department of Infection Biology and Microbiomes, Institute of Infection, Veterinary and Ecological Sciences, University of Liverpool, Liverpool Science Park IC2, 146 Brownlow Hill, Liverpool, L3 5RF, UK; b College of Veterinary Medicine, University of Al-Qadisiyah, Qadisiyah Province, Iraq; c Department of Biology, University of Antwerp, Universiteitsplein 1, 2610, Wilrijk, Belgium; d Department of Ecology and Evolutionary Biology, Princeton University, M26 Guyot Hall, Princeton, NJ, 08544, USA; e Interuniversity Institute for Biostatistics and Statistical Bioinformatics, Hasselt University, Agoralaan Building D, 3590, Diepenbeek, Belgium; f Centre of Rickettsiosis and Arthropod-Borne Diseases, Hospital Universitario San Pedro-CIBIR, C/ Piqueras, 98, Logroño, 26006, La Rioja, Spain

**Keywords:** *Candidatus* Rickettsia vini, *Ixodes arboricola*, Tree-hole tick, Tick cell line, Endosymbiont, *Parus major*

## Abstract

*Candidatus* Rickettsia vini was originally detected in *Ixodes arboricola* ticks from Spain, and subsequently reported from several other Western Palearctic countries including Belgium. Recently, the bacterium was isolated in mammalian (Vero) cell culture from macerated male *I. arboricola* from Czech Republic, but there have been no reports of propagation in tick cells. Here we report isolation in a tick cell line of three strains of *Ca.* R. vini from *I. arboricola* collected from nests of great tits (*Parus major*) in Belgium. Internal organs of one male and two engorged female ticks were dissected aseptically, added to cultures of the *Rhipicephalus microplus* cell line BME/CTVM23 and incubated at 28 °C. *Rickettsia*-like bacteria were first seen in Giemsa-stained cytocentrifuge smears between 2 and 15 weeks later. Two of the isolates grew rapidly, destroying the tick cells within 2–4 weeks of onward passage in BME/CTVM23 cells, while the third isolate grew much more slowly, only requiring subculture at 4−5-month intervals. PCR amplification of bacterial 16S rRNA and *Rickettsia gltA*, *sca4, ompB*, *ompA* and 17-kDa genes revealed that all three isolates were *Ca.* R. vini, with 100 % identity to each other and to published *Ca.* R. vini sequences from other geographical locations. Transmission electron microscopy revealed typical single *Rickettsia* bacteria in the cytoplasm of BME/CTVM23 cells. The *Ca.* R. vini strain isolated from the male *I. arboricola* tick, designated Boshoek1, was tested for ability to grow in a panel of *Ixodes ricinus*, *Ixodes scapularis* and *R. microplus* cell lines and in Vero cells. The Boshoek1 strain grew rapidly, causing severe cytopathic effect, in the *R. microplus* line BME26, the *I. ricinus* line IRE11 and Vero cells, more slowly in the *I. ricinus* line IRE/CTVM19, possibly established a low-level infection in the *I. ricinus* line IRE/CTVM20, and failed to infect cells of any of four *I. scapularis* lines over a 12-week observation period. This study confirmed the applicability of the simple tick organ-cell line co-cultivation technique for isolation of tick-borne *Rickettsia* spp. using BME/CTVM23 cells.

## 1 Introduction

*Ixodes arboricola*, a nidicolous tick species commonly parasitizing cavity-nesting birds such as great tits (*Parus major*) and blue tits (*Cyanistes caeruleus*), is distributed throughout Palearctic Europe, from the Iberian Peninsula in the west, UK and Sweden in the north, European parts of Russia in the east, through to Turkey (Heylen et al., 2014a; Keskin et al., 2014; Novakova et al., 2015; Palomar et al., 2015; Van Oosten et al., 2018). This tick has been reported to harbour bacteria from five different genera – *Borrelia*, *Rickettsia*, *Spiroplasma*, *Rickettsiella* and *Midichloria* (Spitalska et al., 2011; Palomar et al., 2012a,b, 2015; Heylen et al., 2013, 2014b; Keskin et al., 2014; Novakova et al., 2015, 2016; Duron et al., 2017; Van Oosten et al., 2018). Of these bacteria, only the species originally designated *Candidatus* Rickettsia vini (Palomar et al., 2012b) has been isolated from *I. arboricola* into mammalian cells and partially characterised; three isolates were propagated through at least four passages *in vitro* in Vero cells, and found to have 100 % identical sequences for fragments of the *gltA*, *ompA*, *ompB* and 17-kDa genes (Novakova et al., 2016). To date, there has been no report of isolation or cultivation of *Ca.* R. vini in tick cells. Availability of tick cell-isolated bacteria would facilitate comparative *in vitro* study of interactions between *Ca.* R. vini, other endosymbiotic and pathogenic *Rickettsia* spp. and cells derived from vector tick genera.

Currently there are no cell lines available from *I. arboricola*; however, certain cell lines derived from other tick species, including *Ixodes ricinus* and *Ixodes scapularis*, have been shown to be highly susceptible to rickettsial infection (Policastro et al., 1997; Munderloh et al., 1998; Simser et al., 2002; Kurtti et al., 2005, 2015). In the present study, organs from *I. arboricola* ticks were inoculated into cultures of the *Rhipicephalus microplus* cell line BME/CTVM23, previously found to be highly susceptible to infection with tick-borne bacteria (Alberdi et al., 2012; Ferrolho et al., 2016; Palomar et al., 2019), in an attempt to isolate any of the bacteria reported to be harboured by this tick species. Here we report isolation, prolonged *in vitro* propagation in a tick cell line, and partial molecular and morphological characterisation of three strains of *Ca.* R. vini.

## 2 Materials and methods

### 2.1 Ticks

The *I. arboricola* ticks used in this study originated from the Boshoek study area (51°07'27"N, 4°31'20"E), approximately 15 km south-east of Antwerp in Belgium. Engorged female ticks and a single male tick, presumed to be unfed as male *I. arboricola* do not normally feed (Van Oosten et al., 2018), were collected in May 2018 from the underside of the lids of wooden nest boxes where great tits (*Parus major*) were rearing their nestlings. The ticks were held in constant darkness in a climate chamber (20 °C, 85 % relative humidity) at the University of Antwerp for 25–36 days. Two engorged female and one male *I. arboricola* were then shipped to the Tick Cell Biobank at the University of Liverpool where they were surface-sterilised by immersion for 3−5 min in 0.1 % benzalkonium chloride and 1 min in 70 % ethanol, rinsed in sterile deionised water and air-dried. The female ticks were incubated in sterile petri dishes for oviposition, while the male was embedded in sterile wax and dissected under Hanks balanced salt solution (HBSS) to obtain its internal organs as described previously (Palomar et al., 2019). Following oviposition, the female ticks were dissected in HBSS as above to obtain their internal organs.

### 2.2 Tick cell lines

Nine embryo-derived tick cell lines were used in the study; their origins and culture media and conditions are shown in Table 1. The *R. microplus* cell line BME/CTVM23 (Alberdi et al., 2012) and *I. ricinus* cell line IRE/CTVM19 (Bell-Sakyi et al., 2007) were used for bacterial isolation, while the other seven cell lines were tested for ability to support growth of isolated bacteria. All bacteria-infected cultures were maintained in 2.2 mL culture medium with antibiotics (100 units/mL penicillin and 100 μg/mL streptomycin, Sigma) in sealed, flat-sided culture tubes (Nunc) in ambient air in a dry incubator at 28 °C, with weekly medium change (removal and replacement of ¾ medium volume).

**Table 1:** Tick cell lines used in the study: their species origin and culture medium, the purpose for which they were used in this study and their original reference.

Cell line	Tick species	Culture medium	Purpose	Reference
BME/CTVM23	*Rhipicephalus microplus*	L-15^1^	Isolation and passage	Alberdi et al. (2012)
BME26	*R. microplus*	L-15B300^2^	Infectivity	Kurtti et al. (1988)
IRE/CTVM19	*Ixodes ricinus*	L-15^3^	Isolation and infectivity	Bell-Sakyi et al. (2007)
IRE/CTVM20	*I. ricinus*	L-15/L-15B^4^	Infectivity	Bell-Sakyi et al. (2007)
IRE11	*I. ricinus*	L-15B300	Infectivity	Simser et al. (2002)
IDE2	*Ixodes scapularis*	L-15B300	Infectivity	Munderloh et al. (1994)
IDE8	*I. scapularis*	L-15B	Infectivity	Munderloh et al. (1994)
ISE6	*I. scapularis*	L-15B300	Infectivity	Kurtti et al. (1996)
ISE18	*I. scapularis*	L-15B300	Infectivity	Munderloh et al. (1994)

1 L-15 (Leibovitz) medium supplemented with 10 % tryptose phosphate broth (TPB), 20 % foetal bovine serum (FBS), 2 mM L-glutamine (L-glut).

2 L-15B medium (Munderloh and Kurtti, 1989) supplemented with 20 % ultrapure water, 10 % TPB, 10 % FBS, L-glut and 0.1 % bovine lipoprotein concentrate (MP Biomedicals).

3 L-15B medium as above but without the 20 % ultrapure water.

4 A 1:1 mixture of L-15 and L-15B media.

### 2.3 Isolation of bacteria

The dissected internal organs of the three *I. arboricola* ticks, comprising parts of midguts, salivary glands, Malpighian tubules, rectal sac, brain, fat body and reproductive organs, were rinsed once in HBSS and inoculated without further treatment into a single culture of BME/CTVM23 cells for each female tick, and one culture each of IRE/CTVM19 and BME/CTVM23 cells for the male tick. The cultures, initially containing approximately 2 × 10^6^ (BME/CTVM23) or 1 × 10^6^ (IRE/CTVM19) cells, were incubated at 28 °C with weekly medium change and visual examination by inverted microscope. At intervals of 2–5 weeks, commencing 2–3 weeks post inoculation (p.i.), Giemsa-stained cytocentrifuge smears were prepared from resuspended cells as described previously (Alberdi et al., 2012) and examined for presence of intracellular bacteria. When bacteria became numerous (either >10 % cells infected, or appearance of cytopathic effect [CPE] manifest as dying cells, or both), 0.1–0.4 mL aliquots of culture supernatant containing a few cells were transferred to fresh naïve BME/CTVM23 cell cultures. Infected BME/CTVM23 cells were cryopreserved in vapour-phase liquid nitrogen as described previously (Palomar et al., 2019).

### 2.4 Molecular analysis

Following centrifugation of 500 μL of resuspended cell culture at 13,000 × g for 10 min at room temperature, DNA was extracted from the resultant pellet using a DNeasy blood and tissue kit (Qiagen) according to the manufacturer’s instruction for Gram-negative bacteria. DNA extracts were screened for detection of bacterial species using a pan-bacterial PCR assay (primers fD1 and rP2) that amplifies a 1,500-bp fragment of the 16S rRNA gene (Weisburg et al., 1991). The samples that yielded positive results with the pan-bacterial PCR were also analysed for presence of bacteria using specific PCR assays for detection of *Rickettsia*, *Spiroplasma*, *Rickettsiella* and *Midichloria* spp. *Rickettsia* isolates were genetically characterized using PCR assays that amplify fragments of the citrate synthase (*gltA*) gene and the genes encoding the 190-kDa protein antigen (*ompA*), the 120-kDa protein antigen (*ompB*), the PS120 protein (*sca4*) and the 17-kDa antigen (17-kDa), as described by the respective authors (Jado et al., 2007; Regnery et al., 1991; Roux et al., 1996; Roux and Raoult, 2000; Sekeyova et al., 2001; Oliveira et al., 2002; Labruna et al., 2004; Choi et al., 2005; Jiang et al., 2013). The *Spiroplasma* analysis was performed using a PCR that amplifies a fragment of the RNA polymerase beta subunit (*rpoB*) (Haselkorn et al., 2009). The samples were tested for *Rickettsiella* and *Midichloria* by, respectively, amplification of a fragment of the gene encoding the chaperon protein GroEL (*GroEL*) (Duron et al., 2015) and a quantitative PCR targeting the gyrase subunit B (*gyrB*) gene (Al-Khafaji et al., 2019).

A negative control containing water instead of template DNA was included in all PCR assays. *Borrelia spielmanii* DNA, kindly provided by Dr Volker Fingerle (German National Reference Centre for *Borrelia*) to the Centre of Rickettsiosis and Arthropod-Borne Diseases, was included in all the 16S rRNA pan-bacterial PCR assays as a positive control. Positive controls were also included in the *Rickettsia*-specific PCR assays: DNA from *Rickettsia amblyommatis* (Santibáñez et al., 2017) or *Rickettsia raoultii* (Palomar et al., 2019). *Spiroplasma* sp. strain Bratislava and a *Rickettsiella* sp. from a tick extract were used as positive controls in the *Spiroplasma* and *Rickettsiella* analyses respectively (Bell-Sakyi et al., 2015; Portillo et al., 2017). The *Midichloria* qPCR employed standards comprising synthetic long oligonucleotides of the full-length amplicon of the *gyrB* gene (Al-Khafaji et al., 2019) as controls.

### 2.5 Sequence and phylogenetic analysis

PCR amplicons of the expected size were purified using a Monarch PCR Product Purification kit (New England Biolabs) following the manufacturer’s instructions. Purified amplification products were sequenced in the forward and reverse directions, and homology searches were performed in the NCBI database using the BLAST search programme (http://blast.ncbi.nlm.nih.gov/Blast.cgi). Sequences were aligned using the European Bioinformatics Institute multisequence software Clustal Omega (https://www.ebi.ac.uk/Tools/msa/clustalo/) for multiple sequence alignment. The resultant sequences were submitted to GenBank through BankIt (https://www.ncbi.nlm.nih.gov/WebSub/). Phylogenetic analyses were conducted with MEGA version X (www.megasoftware.net) using the maximum likelihood method and Kimura 2-parameter model and including all sites, with all positions containing gaps and missing data included for analysis (by setting “use all sites” in the “Gaps/Missing data treatment” option). Confidence values for individual branches of the resulting trees were determined by bootstrap analysis with 500 replicates. The published sequences used in the analyses are shown in the phylogenetic tree.

### 2.6 Transmission electron microscopy of *Ca.* R. vini -infected tick cells

Resuspended cells from a *Ca.* R. vini -infected BME/CTVM23 culture were centrifuged at 200 × *g* for 5 min, washed once in PBS, fixed in 2.5 % glutaraldehyde (w/v) in 0.1 M phosphate buffer (0.08 M Na_2_HPO_4_, 0.02 M NaH_2_PO_4_, pH 7.4) in a Pelco Biowave (Ted Pella Inc.). The cells were then washed three times in 0.1 M phosphate buffer before being embedded in 3 % agarose, set on ice and cut into small cubes. The cubes were then post-fixed and stained with 2 % aqueous osmium tetroxide in the Pelco Biowave, followed by 2 % aqueous uranyl acetate overnight at 4 °C. To prevent precipitation artifacts, the cubes were washed for a minimum of 5 × 3 min with double-distilled water (ddH_2_O) between each staining step. The cubes were then washed in ddH_2_O before dehydration in a graded series of acetone at 30 %, 50 %, 70 % and 90 % in ddH_2_O for 5 min each, followed by 2 × 5 min in 100 % acetone. Samples were then infiltrated with medium Premix resin (TAAB) at 30 %, 50 % and 75 % resin in acetone for 30 min each, followed by 3 × 100 % resin steps for 30 min each. Fresh 100 % resin was used to embed pellets in silicone moulds before being cured for 48 h at 60 °C. Ultrathin serial sections (70−75 nm) were cut on an UC6 ultramicrotome (Leica, Vienna) and collected on formvar coated copper grids. Grids were post-stained with 4% uranyl acetate and lead citrate, before viewing at 120KV in a FEI Tecnai G2 Spirit transmission electron microscope (FEI, Hillsboro, Oregon, USA). Images were taken with a Gatan RIO16 camera (Gatan, Pleasanton, USA) using GMS3 software.

### 2.7 Infectivity of *Ca.* R. vini for other *R. microplus* and *Ixodes* spp. cell lines and Vero cells

Supernatant medium from BME/CTVM23 cultures heavily infected with *Ca.* R. vini (>80 % of cells undergoing lysis determined by inverted microscopy) was centrifuged at 1,500 x g for 5 min to remove intact tick cells and then inoculated as 0.3 mL aliquots into one tube per cell line of each of the nine tick cell lines listed in Table 1. All tick cell cultures were incubated at 28 °C for up to 12 weeks p.i. and Giemsa-stained cytocentrifuge smears were examined for presence of intracellular bacteria at 2–4 week intervals. DNA extracted from cultures visually negative for *Rickettsia* at 12 weeks p.i. was screened using the *Rickettsia gltA* PCR assay as described above. Tick cell-free supernatant from a BME/CTVM23 culture infected with *Ca.* R. vini was also added to cultures of Vero cells (ECACC cat. no. 84113001) maintained in L-15 (Leibovitz) medium supplemented with 5% foetal bovine serum and antibiotics as above, in sealed flat-sided culture tubes at 32 °C; subcultures onto fresh Vero cells were carried out at two-weekly intervals by transfer of 0.2–0.3 mL of cell-free supernate.

## 3 Results

**Fig. 1:**
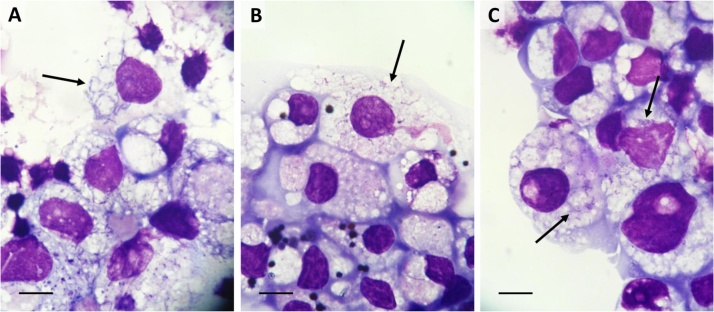
**Light micrographs of *Candidatus* Rickettsia vini isolated from Belgian *Ixodes arboricola* ticks into cells of the *Rhipicephalus microplus* tick cell line BME/CTVM23. A**: Boskoek1 isolate at passage 10, one year after initial isolation; **B**: Boshoek2 isolate in parent culture, 44 days after initial isolation; **C**: Boshoek3 isolate in parent culture, 211 days after initial isolation. Giemsa-stained cytocentrifuge smears, arrows indicate infected cells, scale bars =10 μm.

Each of the three ticks whose organs were inoculated into a BME/CTVM23 culture yielded an isolate of intracytoplasmic gram-negative, rod-shaped bacteria with the size and appearance of *Rickettsia* (Fig. 1), while no bacteria were detected in the IRE/CTVM19 culture inoculated with organs from the male tick over the subsequent 6 months. Initial pan-bacterial 16S rRNA gene PCR and sequence analysis confirmed the presence of a *Rickettsia* sp. in all three cultures. The three nucleotide sequences, with a size of 1,367 bp, were identical and the blast analysis showed a maximum identity of 99.85 % with *Rickettsia japonica* (AP017602). The cultures were also screened with genus-specific PCR assays for *Spiroplasma*, *Rickettsiella* and *Midichloria*, as these bacterial genera have been detected in *I. arboricola* (Duron et al., 2017; Van Oosten et al., 2018), but no PCR products were amplified. Isolation of *Borrelia* spp. was precluded by inclusion of antibiotics in the culture media.

The three *Rickettsia* sp. isolates obtained from the male tick and the two female ticks were designated Boshoek1, Boshoek2 and Boshoek3 respectively, and were further characterised to identify the species. The sequences corresponding to each rickettsial gene obtained from the three isolates were identical to each other and homologous (100 % identical) to *Ca.* R. vini sequences published in GenBank (16S rRNA: 396 bp, JF758824; *gltA*: 1,068 bp, JF803266; *sca4*: 888 bp, JF758829; *ompB*: 455 bp, JF758826; *ompA*: 590 bp, JF758828; 17-kDa: 394 bp, JF758827; Palomar et al., 2012b). In the analysis of the 16S rRNA, *ompB* and *ompA* nucleotide sequences, the query cover did not reach 100 % because the publicly-available sequences of *Ca.* R. vini are shorter than those obtained in the present study. These new sequences have been deposited in GenBank under the following accession numbers: MT062903 (*sca4*), MT062904 (16S rRNA), MT062905-6 (*ompB*), MT062907 (*ompA*), MT062908 (17-kDa) and MT062909 (*gltA*).

**Fig. 2:**
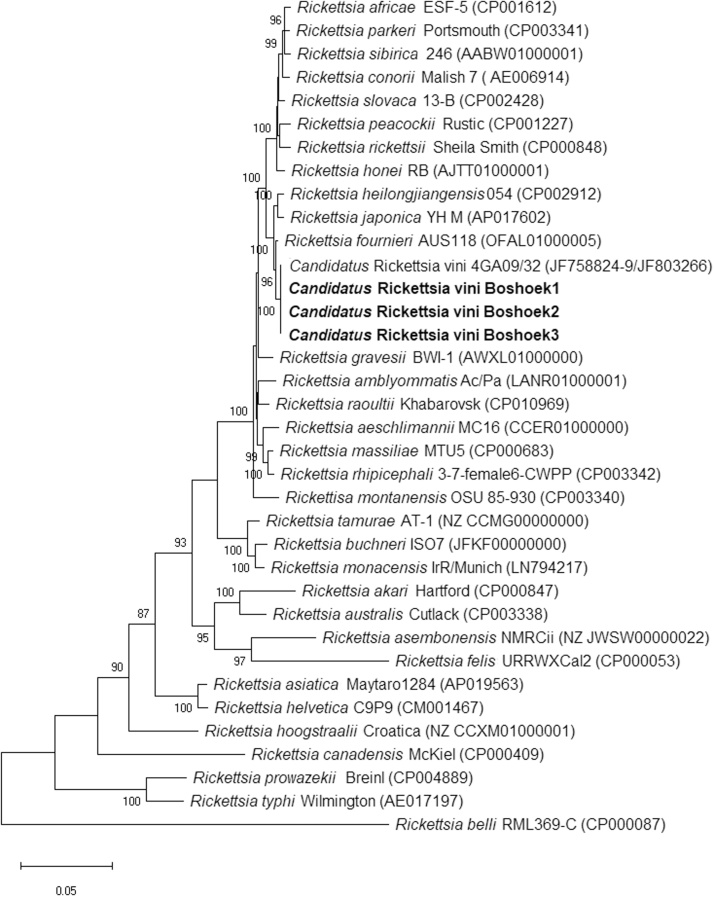
**Phylogenetic tree showing the relationships between three *Candidatus* Rickettsia vini isolates obtained from Belgian *Ixodes arboricola* ticks and grown in the tick cell line BME/CTVM23 (in bold), and published *Rickettsia* spp. sequences.** The evolutionary analysis was inferred using the maximum likelihood method and Kimura 2-parameter model within the Mega X software, by concatenating fragments of six genes (*sca4*, 16 s rRNA, *ompB*, *ompA*, 17-kDa and *gltA*). This analysis involved 36 nucleotide sequences and gaps and missing data were included in the analysis, a total of 5,900 positions in the final dataset. The scale bar represents a 5% estimated difference in nucleotide sequence. Numbers shown at the nodes correspond to the percentage bootstrap values (for 500 repetitions). Replicate numbers of <85 % are not shown. The GenBank accession numbers of the sequences used in this analysis are shown in brackets following each *Rickettsia* species and the corresponding strain.

The phylogenetic tree constructed from the concatenated gene sequences shows the position of the three Boshoek isolates as homologous to *Ca.* R. vini and closely related to *Rickettsia fournieri*, *Rickettsia heilongjiangensis* and *R. japonica* (Fig. 2).

Two of the isolates, Boshoek1 and Boshoek2, first became patent at 4 weeks p.i. and the first passage onto fresh BME/CTVM23 cells became necessary at 19 weeks due to destruction of the majority of cells in the initial culture. Subsequent passage intervals became progressively shorter, so that by passage 6, the bacteria were destroying the majority of tick cells (>80 % cells undergoing lysis) within two weeks following a 1 in 20 dilution. Aliquots of these two isolates were cryopreserved as the parent culture or at passage 1 respectively, 26 weeks after initial isolation, and later at passage levels 10 and 7 respectively. The third isolate, Boshoek3, grew much more slowly than Boshoek1 and Boshoek2; the bacteria were first detected in Giemsa-stained smears at 15 weeks p.i. Despite absence of any obvious CPE the first subculture was carried out at 22 weeks p.i., and the parent culture was cryopreserved at 30 weeks p.i. In contrast to the Boshoek1 and Boshoek2 isolates, that caused severe CPE in their parent cultures, the Boshoek3 isolate did not cause any detectable CPE until passage 2, at 39 weeks after the initial inoculation.

**Fig. 3:**
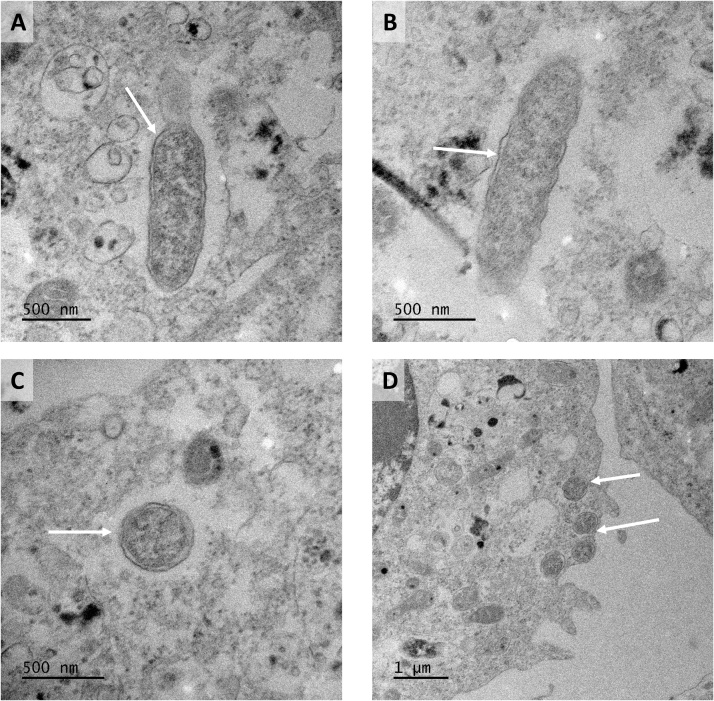
**Transmission electron micrographs of BME/CTVM23 cells infected with the Boshoek1 isolate of *Candidatus* Rickettsia vini. A-C**: single bacteria (arrows) in the cytoplasm of tick cells (scale bars =500 nm); **D**: multiple bacteria (arrows) in the cytoplasm of a single tick cell (scale bar =1 μm). Photomicrographs captured with a Gatan RIO16 camera using GMS3 software.

Transmission electron microscopy revealed the presence of numerous rod-shaped bacteria in the cytoplasm of BME/CTVM23 cells infected with the Boshoek1 strain at passage 10, 14 months after isolation (Fig. 3). The bacteria were typical of *Rickettsia* spp.: single organisms with mottled cytoplasm bound by an inner periplasmic membrane, an electron-lucent periplasmic space and an outer rippled cell wall, sometimes surrounded by an electron-lucent halo (Fig. 3A, B, C), and often located near the periphery of the cell (Fig. 3D). The bacteria measured between 1.21 and 1.41 μm in length and between 0.27 and 0.40 μm in cross-sectional diameter.

**Fig. 4:**
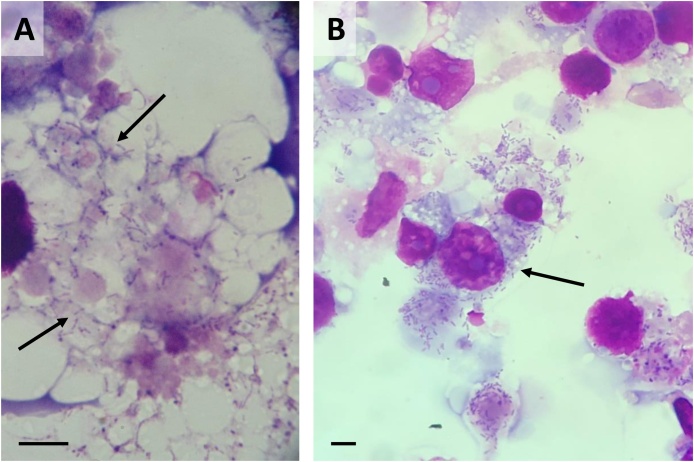
**Infectivity of *Candidatus* Rickettsia vini (Boshoek1 strain) for cell lines other than BME/CTVM23. A**: *Ca.* R. vini in the cytoplasm of a cell of the *Ixodes ricinus* cell line IRE/CTVM19 60 days after transfer from BME/CTVM23 cells. B. *Ca.* R. vini at passage 3 in Vero cells grown at 32 °C, 46 days after transfer from BME/CTVM23 cells. Giemsa-stained cytocentrifuge smears, scale bars =10 μm.

The Boshoek1 isolate was selected for infectivity testing with additional tick cell lines; these studies were observational in nature and quantitative assessments were not conducted. Following inoculation of cell-free culture supernate from BME/CTVM23 cells infected with *Ca.* R. vini at passage 6, intracellular *Rickettsia* were first detected in BME/CTVM23 (control culture), *R. microplus* BME26 cells and *I. ricinus* IRE11 cells at 2, 2 and 4 weeks p.i. respectively, and the cultures succumbed to a heavy infection at 3, 4 and 6 weeks p.i. respectively. The IRE/CTVM19 culture became detectably infected at 8 weeks p.i., but did not succumb to infection despite presence of large numbers of intracellular bacteria (Fig. 4A). By 12 weeks p.i., the remaining cell lines (*I. ricinus* IRE/CTVM20 and the four *I. scapularis* lines IDE2, IDE8, ISE6 and ISE18) did not show any CPE or signs of *Rickettsia* infection detectable by microscopy. PCR amplification of a fragment of the *gltA* gene was attempted from these five cultures; the four *I. scapularis* cell lines were all negative. However, the IRE/CTVM20 culture yielded a positive PCR product confirmed as *Ca.* R. vini by sequencing.

Infectivity of *Ca.* R. vini for Vero cells maintained at 32 °C was also tested, using the Boshoek1 isolate at passage 10 in BME/CTVM23 cells. Within two weeks, a heavy infection had developed (Fig. 4 B), with CPE evident as detached and dying cells undergoing lysis. The bacteria were taken through four passages in Vero cells with no change in the time taken to induce CPE.

The Boshoek1, Boshoek2 and Boshoek3 isolates of *Ca.* R. vini are deposited as cryopreserved stabilates of infected BME/CTVM23 cell cultures in the Tick Cell Biobank at the University of Liverpool.

## 4 Discussion

A novel *Rickettsia* species infecting *Ixodes* spp. ticks removed from birds in La Rioja, Spain was first described and partially characterised as *Candidatus* Rickettsia vini by Palomar et al. (2012a, 2012b). *Ca.* R. vini was subsequently found to be widespread in European and Near Eastern *I. arboricola* and *Ixodes lividus* ticks (Keskin et al., 2014; Nováková et al., 2015, 2016, 2018; Palomar et al., 2015; Van Oosten et al., 2018; Matulaityte et al., 2020), and the bacterium was first isolated from *I. arboricola* from Czech Republic into Vero cells by Novakova et al. (2016). Phylogenetically, the most closely related bacterium is the recently-described *R. fournieri* (Diop et al., 2018), a species isolated from the soft tick *Argas lagenoplastis* in Australia, whose pathogenicity for humans is unknown. *Ca.* R. vini is also closely related to the spotted fever group species *R. japonica* and *R. heilongjiangensis*, both of which are highly pathogenic in humans and small laboratory animals (Uchida, 1993; Jiao et al., 2005; Meng et al., 2015). In contrast, Novakova et al. (2016) demonstrated absence of any clinical response following inoculation of guinea pigs and chickens with their cultured *Ca.* R. vini, and infestation of chickens with infected *I. arboricola* larvae. It will be important to obtain full genome sequences from multiple cultured *Ca.* R. vini isolates, to allow a fuller comparison with those *Rickettsia* species that are genetically closely-related but of widely-differing pathogenicity.

Belgian *I. arboricola* ticks were previously reported to harbour *Ca.* R. vini DNA at a prevalence of nearly 100 % (90/93 ticks infected, Van Oosten et al., 2018), suggesting that it may be a tick endosymbiont. In addition, much lower proportions of ticks in the same study harboured DNA of the genera *Rickettsiella* (19/93) and *Spiroplasma* (13/93). These relative prevalences were reflected in the present study, in which we isolated *Ca.* R. vini from all three sampled ticks (one male and two engorged females), but did not detect evidence for *Spiroplasma* or *Rickettsiella* in any of the inoculated cultures. High prevalence of *Ca.* R. vini in *I. arboricola* was also reported in partly-fed larvae, nymphs and adult females removed from birds in Spain (94.4 %, Palomar et al., 2015), and in partly-fed nymphs and adult females removed from nestlings, and unattached nymphs, males and females removed from nest boxes, in Czech Republic and Slovakia (98.2 %, Novakova et al., 2015). These authors did not investigate prevalence of *Spiroplasma* or *Rickettsiella* in the ticks that they tested.

Our study confirms the tick organ-cell line co-cultivation technique, first used for successful isolation of strains of *Anaplasma phagocytophilum* that could not be isolated into mammalian cells (Massung et al., 2007), as a simple method with minimal intervention, and BME/CTVM23 as a highly permissive cell line useful for isolating and propagating tick-borne bacteria. Previously these cells have been used to isolate *R. raoultii*, *Rickettsia slovaca* and *Spiroplasma* (Palomar et al., 2019), and to propagate *Ehrlichia canis* and *Ehrlichia ruminantium* (Ferrolho et al., 2016), as well as *Ehrlichia minasensis*, *Rickettsia buchneri* and *Rickettsia peacockii* (Bell-Sakyi, unpublished results). Another *R. microplus* cell line, BME26 (Kurtti et al., 1988) was also highly permissive for *Ca.* R. vini; this line was also highly susceptible to infection with *R. peacockii* (Kurtti et al., 2005). Of the three *I. ricinus* cell lines tested, IRE11 was highly permissive, with destruction of the culture within 4 weeks, whereas IRE/CTVM19 appeared to tolerate *Ca.* R. vini infection in the absence of CPE over 12 weeks. In IRE/CTVM20, bacteria were not detectable by microscopy, but the more sensitive *gltA* PCR assay indicated presence of a possible low-level *Ca.* R. vini infection at 12 weeks p.i. In contrast, none of the four *I. scapularis* cell lines tested became detectably infected with *Ca.* R. vini. Further studies involving sub-inoculation and quantitative analysis are needed to definitively determine the susceptibility of *Ixodes* spp. cell lines to *Ca.* R. vini infection. Variation in levels of rickettsial infection between different *I. scapularis* cell lines has been reported previously; Kurtti et al. (2005) found that ISE6 cells were highly susceptible to *R. peacockii* infection, while the bacteria could be maintained as chronic infections in IDE2 and IDE8 cells, and Munderloh et al. (1998) found that both IDE2 and IDE8 cells were destroyed within a week by a spotted fever group *Rickettsia* isolated from *Amblyomma americanum* ticks.

Ultrastructurally, *Ca.* R. vini isolated and propagated in tick cells resembled other *Rickettsia* spp. grown in BME/CTVM23 cells (Alberdi et al., 2012) and other tick cell lines such as IDE2 (Policastro et al., 1997; Munderloh et al., 1998), ISE6 (Simser et al., 2002; Pornwiroon et al., 2006) and IRE11 (Kurtti et al., 2015). *Ca.* R. vini appeared as individual bacteria free in the host cell cytoplasm; vacuoles containing multiple bacteria, as reported by Pornwiroon et al. (2006) for *Rickettsia felis* in ISE6 cells and Kurtti et al. (2015) for *R. buchneri* in IRE11 cells, were not seen.

There is only one previous report of isolation of *Ca.* R. vini from macerated, whole male *I. arboricola* ticks, using the shell vial technique with Vero cells grown at 28 °C (Novakova et al., 2016). These authors did not report the duration of the incubation period until *Ca.* R. vini was detectable by microscopy or molecular analysis, or the passage interval in Vero cells, so it is not possible say whether, in general, the bacterium adapts more quickly to, and grows faster in, tick or mammalian cell culture. However, in the present study a wide variation in prepatent period of between 2 and 15 weeks was observed in tick cells, suggesting that isolates of *Ca.* R. vini may differ in their growth rate or ability to adapt to *in vitro* cultivation. Our Boshoek1 isolate first became patent at 4 weeks post inoculation in BME/CTVM23 cells at 28 °C, but within 6 passages it was destroying the majority of cells within two weeks, and when transferred to Vero cells grown at 32 °C, this rate of induction of CPE was maintained.

As well as *Ca.* R. vini, *I. arboricola* ticks are known to harbour *Spiroplasma*, *Rickettsiella*, *Midichloria* and *Borrelia* spp., but these bacteria have been reported to occur at lower prevalence (Heylen et al., 2013; Duron et al., 2017; Van Oosten et al., 2018). *Spiroplasma* were previously isolated easily from *I. ricinus* and *Dermacentor* spp. ticks (Bell-Sakyi et al., 2015; Palomar et al., 2019) using BME/CTVM23 and other tick cell lines included in the present study, so if *Spiroplasma* were present in any of the *I. arboricola* inocula, it is likely that they would have been isolated. Isolation of *Rickettsiella* has not so far been achieved from *Ixodes* spp. ticks, although Thu et al. (2019) reported CPE accompanying presence of *Rickettsiella* DNA in ISE6 cells inoculated with homogenised male Japanese *Haemaphysalis concinna* ticks. Their study was only continued for 8 weeks, and recognisable bacteria were not detected in Giemsa-stained smears, so it is unclear whether or not *Rickettsiella* became established in the ISE6 cells, and other possible reasons for the CPE were not investigated. To date, there have not been any reports of successful isolation or propagation of *Midichloria* in tick cell lines; although Najm et al. (2011) described presence of a small fragment of the 16S rRNA gene of *Midichloria* in the tick cell lines IRE/CTVM19 and *Rhipicephalus decoloratus* BDE/CTVM14, both cell lines were PCR-negative for a larger fragment of the same gene. While prevalences of *Borrelia* spp. infection of questing adult *I. arboricola* may reach nearly 11 % (Heylen et al., 2013), use of antibiotics, specifically penicillin, in the present study precluded isolation of any spirochaetes that might have been harboured by the parent ticks.

In conclusion, availability of the three isolates of *Ca.* R. vini from Belgian ticks reported here, in addition to the three *Ca.* R. vini isolates previously cultured from ticks from Czech Republic (Novakova et al., 2016), will facilitate further study of this microorganism, in particular genome sequencing and comparison with other *Rickettsia* species, both pathogens and endosymbionts.

## CRediT authorship contribution statement

**Alaa M. Al-Khafaji:** Investigation, Formal analysis, Writing - review & editing. **Lesley Bell-Sakyi:** Conceptualization, Resources, Investigation, Writing - original draft, Writing - review & editing. **Gerardo Fracasso:** Resources, Writing - review & editing. **Lisa Luu:** Investigation, Formal analysis, Writing - review & editing. **Dieter Heylen:** Supervision, Writing - review & editing. **Erik Matthysen:** Supervision, Writing - review & editing. **José A. Oteo:** Formal analysis, Writing - review & editing. **Ana M. Palomar:** Conceptualization, Investigation, Formal analysis, Writing - original draft, Writing - review & editing.

## Declaration of Competing Interest

The authors have no competing interests to declare.
